# Effect of Osteoarthritis on Prefrontal Cortical Activation Patterns During Downward Reaching in Older Women

**DOI:** 10.1093/geroni/igaa057.392

**Published:** 2020-12-16

**Authors:** Manuel Hernandez, Yang Hu, Alka Bishnoi

**Affiliations:** University of Illinois at Urbana-Champaign, Urbana, Illinois, United States

## Abstract

Downward reaching may lead to falls in older adults, but the underlying mechanisms are poorly understood, particularly in older women with osteoarthritis. Given the importance of attentional resources when maintaining balance in balance-demanding conditions, functional near-infrared spectroscopy may provide a lens to the attentional resource allocation needed to maintain balance while reaching down to the ground with or without full contact with the floor. We examined the changes in the executive control of downward reaching movements in older women with osteoarthritis. We hypothesized that prefrontal cortical activation would be higher in older women with osteoarthritis, compared to age-matched controls, particularly as the balance demands increased. Older women with osteoarthritis (n=7, mean±SD age: 66±3 years) and age-matched controls (n=10, mean±SD age: 67±6 years) were recruited from the local community. The effect of the base of support and target position (toe or maximal forward distance along ground) on attentional resources in older women with osteoarthritis were evaluated using the average oxygenated hemoglobin levels as a measure of prefrontal cortical (PFC) activation. Significant base of support by cohort and target by cohort interactions were observed on average PFC activation levels (P<0.005). As expected, PFC activation levels increased as the contact with the floor decreased and as the target distance increased, but older women with osteoarthritis were unable to increase their PFC activation levels as much as age-matched controls. In conclusion, these data suggest that older women with osteoarthritis may not be adequately modulating attentional resources to meet high-balance demanding tasks.

